# Anthocyanins as Antidiabetic Agents—In Vitro and In Silico Approaches of Preventive and Therapeutic Effects

**DOI:** 10.3390/molecules25173813

**Published:** 2020-08-21

**Authors:** Hélder Oliveira, Ana Fernandes, Natércia F. Brás, Nuno Mateus, Victor de Freitas, Iva Fernandes

**Affiliations:** LAQV, REQUIMTE, Departamento de Química e Bioquímica, Faculdade de Ciências, Universidade do Porto, Rua do Campo Alegre s/n, 4169-007 Porto, Portugal; helder.oliveira@fc.up.pt (H.O.); ana.fernandes@fc.up.pt (A.F.); nbras@fc.up.pt (N.F.B.); nbmateus@fc.up.pt (N.M.); vfreitas@fc.up.pt (V.d.F.)

**Keywords:** anthocyanins, anti-diabetic, preventive, therapeutic, microbiota, enzyme, carbohydrate

## Abstract

Many efforts have been made in the past two decades into the search for novel natural and less-toxic anti-diabetic agents. Some clinical trials have assigned this ability to anthocyanins, although different factors like the food source, the amount ingested, the matrix effect and the time of consumption (before or after a meal) seem to result in contradictory conclusions. The possible mechanisms involved in these preventive or therapeutic effects will be discussed—giving emphasis to the latest in vitro and in silico approaches. Therapeutic strategies to counteract metabolic alterations related to hyperglycemia and Type 2 Diabetes Mellitus (T2DM) may include: (a) Inhibition of carbohydrate-metabolizing enzymes; (b) reduction of glucose transporters expression or activity; (c) inhibition of glycogenolysis and (d) modulation of gut microbiota by anthocyanin breakdown products. These strategies may be achieved through administration of individual anthocyanins or by functional foods containing complexes of anthocyanin:carbohydrate:protein.

## 1. Introduction

Diabetes mellitus (DM) is a chronic metabolic disorder characterized by high sugar concentrations in the blood. It may be due to impaired insulin secretion, resistance to peripheral actions of insulin, or both. It can also be accompanied by metabolic abnormalities of lipids, proteins, mineral salts or electrolytes [1]. According to the International Diabetes Federation (IDF), approximately 415 million adults between the ages of 20 to 79 years had diabetes mellitus in 2015 [2]. DM is proving to be a global public health burden as this number is expected to rise to further 200 million by 2040 [2]. Chronic hyperglycemia in synergy with the other metabolic aberrations in patients with diabetes mellitus can cause damage to various organ systems, leading to the development of disabling and life-threatening health complications, most prominent of which are microvascular (retinopathy, nephropathy, and neuropathy) and macrovascular complications leading to increased risk of cardiovascular diseases. DM is rapidly increasing worldwide, powered by the prevalence of unhealthy lifestyles, such as obesity, dietary patterns, sedentarism, alcohol consumption, or smoking [3,4]. With an increase in age, the prevalence of DM also increases. About 25% of the population above 65 years of age has diabetes [5].

DM is generally classified into three types by etiology and clinical presentation, type 1 diabetes, type 2 diabetes, and gestational diabetes (GDM). Since type 2 diabetes mellitus (T2DM) accounts for around 90% of all cases of diabetes, this review will focus on T2DM.

In T2DM, the response to insulin is diminished, and this is defined as insulin resistance. During this state, insulin is ineffective and is initially contradicted by an increase in insulin production to maintain glucose homeostasis, but over time, insulin production decreases, resulting in T2DM. This disease is most commonly seen in persons older than 45 years. Still, it is increasingly seen in children, adolescents, and younger adults, due to rising levels of obesity, physical inactivity, and energy-dense diets.

### 1.1. Diabetes and Related Diseases

#### 1.1.1. Diabetic Peripheral Neuropathy

Although there are many possible causes of peripheral neuropathy, the most prevalent subtype, diabetic peripheral neuropathy (DPN), can lead to major complications [6]. Over half of people with diabetes develop neuropathy, and DPN is a major cause of reduced quality of life due to pain, sensory loss, gait instability, fall-related injury, and foot ulceration and amputation. Early assessment of symptoms of peripheral polyneuropathy helps to avoid neuropathic foot ulcers to ultimately combat potential morbidity and mortality [7]. The exact cause of diabetic peripheral neuropathy is not known. Proposed theories include metabolic, neurovascular, and autoimmune pathways.

#### 1.1.2. Diabetic Retinopathy and Macular Edema

Currently, a major concern of our aging society is to efficiently delay the onset of age-related diseases, which are progressively rising in incidence. Diabetic retinopathy (DR) and age-related macular degeneration (AMD) are retinal degenerative diseases which are leading causes of vision loss and blindness worldwide (WHO 2019). Of an estimated 468 million people with diabetes mellitus worldwide, more than 90 million diabetics have some form of diabetic retinopathy. Due to the increasing prevalence of diabetes, aging of the population, and increased life expectancy of those with diabetes, these numbers are expected to rise. Considering AMD, in 2040, it is estimated that 288 million will have the disease.

Many interconnected mechanisms are implicated in the complex pathogenesis of DR, including oxidative stress [8]. Crosstalk between oxidative stress-related and inflammatory pathways is likely to be important to induce blood-retinal barrier (BRB) breakdown and pathological neovascularization [9]. Disruption of this barrier results in the accumulation of macular edema; however, the process is more complicated than this and also involves various inflammatory markers (such as VEGF, leukocyte adhesion, and protein kinase C) upregulated by advanced glycation end products (AGEs).

The paradigm that a diet rich in phenolics, prevalent in e.g., in fruits, is beneficial to delay age-related diseases has reached the public, especially concerning the awareness of the antioxidant properties of red fruits. Consequently, oxidative stress and inflammatory biomarker inhibitors may have a protective effect on the retina. Antioxidants, in particular anthocyanins, may be considered beneficial to DR because they reduce ROS production and enhance the antioxidant defense system [10].

A better understanding of this dysfunction and the elucidation of the complex crosstalk between nutrition and the initial stages of disease progression may facilitate the development of novel personalized diets with significant clinical benefit in comparison with existing therapies. Simultaneously, chronic interventions addressing multiple metabolic and signaling pathways may be beneficial to attenuate oxidative stress and inflammation and ameliorate the retinal injury.

## 2. Why Anthocyanins?

In trying to find new antidiabetic compounds with fewer side effects, natural sources are being explored [11]. In fact, their consumption in association with trace elements such as chromium or zinc is also receiving attention from researchers. The activation of insulin receptor signaling (chromium), antioxidant properties (selenium, zinc) or inhibition of phosphatases (vanadium) thus appear promising in view of the key importance of these processes in glucose homeostasis and insulin sensitivity [12].

Bioactive-compound-rich foods, including alkaloids, phenols, flavonoids, saponins, polysaccharides, terpenoids, glycosides and xanthones, can be consumed on a daily basis [13].

**Figure 1:**
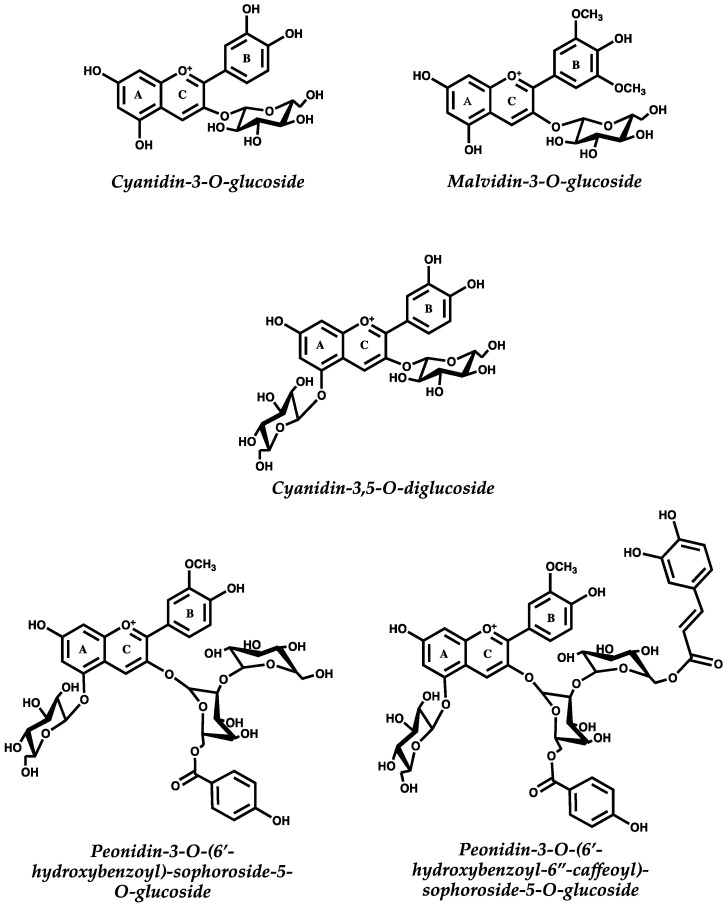
Structure of different anthocyanins (flavylium cation form) commonly found in typical diets. Cyanidin-3-*O*-glucoside can be found in different berries, while cyanidin-3,5-*O*-diglucoside may be found in pomegranates. Malvidin-3-*O*-glucoside is the main anthocyanin in Vitis vinifera grapes and red wine, while sophoroside anthocyanins are abundant in purple sweet potato and purple cabbage.

Anthocyanins (Figure 1), a sub-class of dietary flavonoids, are a large group of water-soluble pigments responsible for many of the colors, ranging red, orange, pink, violet and blue, in fruits, vegetables, flowers and leaves [14,15]. Anthocyanins can be found in daily foods, highlighting berry-like fruits, such as cranberry, chokeberry, blackberry, gooseberry, black grape, bilberry, blueberry, red raspberry, blackcurrant, redcurrant or strawberry, other fruits as red apple, nectarine, peach, plum, radish, pomegranate and cherry; vegetables as red onion, red cabbage, eggplant, purple potatoes, seeds as black beans and beverages as red grape wines or red fruit juices [16]. Anthocyanin content and composition varies greatly in plant foods with berries being the ones with the higher anthocyanin concentration in the diet [17]. 

The human diet includes six main anthocyanidins (aglycone): cyanidin, delphinidin, malvidin, pelargonidin, peonidin, and petunidin. Color is a general guide to which anthocyanins are present, which may be important for dietary guidance as research identifies specific effects of certain anthocyanins and/or their metabolites in T2DM risk reduction. Average daily intake of anthocyanin is ~10.3 mg/day in the USA [18]. However, a higher intake of anthocyanins has been reported in Italy (44.1–64.9 mg/d) [19] and Finland (47 mg/d) [20], possibly due to a higher berry consumption [21] compared with berry intake in the USA [22].

Amongst the several classes of dietary flavonoids, anthocyanins are regarded as the least bioavailable. However, data reported in the literature regarding the bioavailability of anthocyanins may be underestimated due to some singularities of this class of flavonoids. Besides glycosylation, that may also be observed in other types of flavonoids, the unique physical–chemical properties of anthocyanins influence their stability, absorption patterns and metabolic fate, clearly affecting their behavior in vivo. Indeed, conversely to other classes of flavonoids, anthocyanins may occur under different equilibrium structures that may have a different biological influence, depending on the surrounding pH [23]. Moreover, they are also very unstable at neutral pH and physiological temperature, and can interact with proteins or carbohydrates [24].

Poor absorption in the upper part of the gastrointestinal tract, susceptibility to gastrointestinal pH, enzymatic environments and the action of the microbiota may also contribute to the reported low bioavailability of anthocyanins; such features result in the detection a relatively small portion of the initial parent anthocyanins in blood and urine [25,26]. Additionally, even absorbed intact anthocyanins may undergo further metabolization in the liver or kidney [27,28]. After ingestion, anthocyanins may be readily absorbed in the oral or gastric cavity [29]; however, the majority of these compounds are not absorbed in the stomach and small intestine, reaching the colon in their intact form [30]. In fact, the bioavailable forms of anthocyanins in vivo are not exclusively the same as those that occur in food, since they are also largely metabolized, yielding several types of metabolites [31]. These include anthocyanin phase II metabolites (glucuronide, methylated and sulfate) or simpler phenolics, e.g., phenylpropionic, phenylacetic, benzoic acids and m-hydroxyphenylpropionic acids, intact or phase II metabolites, which can be absorbed by enterohepatic recirculation [32,33,34]. So, although anthocyanins present an apparent low bioavailability, their metabolites may play an important role in the in vivo protective effects observed. 

For example, protocatechuic acid, the main gut metabolite of C3G, exhibited a slight inhibitory effect on nitric oxide (NO) production and Tumor necrosis factor alpha (TNF-α) secretion in lipopolysaccharide (LPS)- gamma interferon (INF-γ) -induced macrophages [35]. Also, gallic acid, the gut metabolite of D3G, caused a decrease in the secretion of monocyte chemoattractant protein-1 (MCP-1), intercellular Adhesion Molecule 1 (ICAM-1) and vascular cell adhesion molecule 1 (VCAM-1) in endothelial cells [35].

In addition, the potential antidiabetic properties of intact anthocyanins could also be partly related to their antioxidant and anti-inflammatory activities. Anthocyanins may decrease the levels of reactive oxygen species, increase the enzyme activity of catalase and superoxide dismutase [36], and may influence angiogenesis by decreasing the vascular endothelial cell growth factor (VEGF) level and inhibiting the protein kinase B (Akt) pathway [36]. In particular, anthocyanins interact with the NF-κB and activator protein-1 (AP-1) signal transduction pathways, which respond to oxidative signals and mediate a proinflammatory effect, and the nuclear erythroid 2-related factor 2/antioxidant response element (Nrf2/ARE) pathway and its regulated cytoprotective proteins (Glutathione S-transferase (GST), NADPH quinone oxidoreductase-1 (NQO-1), Heme oxygenase-1 (HO-1), etc.), involved in both cellular antioxidant defense and the elimination/inactivation of toxic compounds, thereby countering the alterations caused by conditions of chemical/oxidative stress [37]. In addition, possible crosstalk could partially explain the protective effects of anthocyanins and their metabolites in diabetes, characterized by an altered balance among these pathways.

However, the concentrations of anthocyanins and their metabolites, when used in in vitro assays, are often many times higher than those that could be physiologically achievable.

### 2.1. Clinical Trials

The available literature on the effect of dietary anthocyanins to reduce the risk of diabetes in human clinical trials is growing.

Twenty-seven overweight or obese men were enrolled in a randomized, placebo-controlled crossover study. Subjects were fed a high-fat diet which contained either 600 g/day blackberries or a calorie and carbohydrate matched amount of gelatin for seven days [38]. Blackberry consumption may promote increased fat oxidation and improved insulin sensitivity in overweight or obese males fed a high fat diet. However, glucose area under the curve (AUC) was not different between the blackberries and gelatin groups [38]. Similarly, consumption of freeze-dried wild blueberry powder in smoothie form did not reduce postprandial glucose levels [39]. Also, consumption of blueberries with a higher carbohydrate meal positively affected the plasma pancreatic polypeptide concentrations which may have an impact on reducing food intake by inducing satiety and meal termination, although glucose metabolism was not significantly altered [40].

Nevertheless, postprandial trials with strawberries suggest intake 2 h after a meal or within 2 h before a meal may be required to attenuated postprandial glucose without additional insulin, providing support for a role of early phase strawberry/berry metabolites in peripheral glucose regulation [41].

However, in a dose-response study in obese individuals with insulin resistance, a beverage containing 40 g of freeze-dried strawberry powder consumed with a high-carbohydrate high-fat meal significantly reduced the post-meal demand for insulin compared to a control beverage devoid of polyphenols, but matched for fiber [42].

Furthermore, statistical evaluation of the dose-dependent strawberry metabolite profiles relative to clinical outcomes indicated an inverse relationship between the primary anthocyanin metabolites of strawberry and insulin responses and glucose clearance [42]. In individuals with pre-diabetes and insulin resistance, intake of 250 g of frozen red raspberry (~2 cups) in a breakfast meal significantly reduced peak and postprandial (2 h) glucose concentrations when compared with the control [43].

Two recent meta-analyses of randomized controlled trials were conducted to investigate the effects of anthocyanin intake/anthocyanin rich foods as sources of anthocyanins on a range of cardiometabolic risk biomarkers. The meta analyses indicated that the effects of anthocyanins significantly reduced fasting glucose, 2-h postprandial glucose, glycated hemoglobin, total cholesterol, low-density lipoproteins (LDL) and blood pressure [44,45], specifically highlighting the beneficial effects of berries as a source of anthocyanins in comparison with red grapes/wine.

In an intervention studies, in people with T2DM, 58 patients were given 160 mg of anthocyanins twice daily or placebo (n = 29/group) for 24 weeks in a randomized, placebo-controlled, double-blind trial [46]. Anthocyanin supplementation exerts beneficial metabolic effects in subjects with type 2 diabetes by improving dyslipidemia, enhancing antioxidant capacity, and preventing insulin resistance compared to the placebo group [46]. Overall, there appears to be complementary data from the collective epidemiological and human clinical trial investigations suggesting that dietary anthocyanins have a strong potential to modulate the risk of T2DM in humans.

In the longest-duration randomized controlled trials, the effect of 6-month blueberry intake on insulin resistance and cardiometabolic function in metabolic syndrome was evaluated in 115 volunteers [47]. A daily intake of 1 cup of blueberries improved endothelial function, systemic arterial stiffness and attenuated cyclic guanosine monophosphate concentrations. In statin nonusers (n = 71), elevated high-density lipoprotein cholesterol, high-density lipoprotein particle density and apolipoprotein A-I concentrations were observed following the 1-cup/d intervention. Treatment compliance was 94.1% (wrapper returns), and total concentrations of anthocyanin-derived phenolic acid metabolites significantly increased, dose-dependently, in serum and 24-h urine. Despite insulin resistance remaining unchanged, the first sustained improvements in vascular function, lipid status, and underlying NO bioactivity following 1 cup blueberries/d were shown. With effect sizes predictive of 12–15% reductions in CVD risk, blueberries should be included in dietary strategies to reduce individual and population CVD risk.

**Figure 2:**
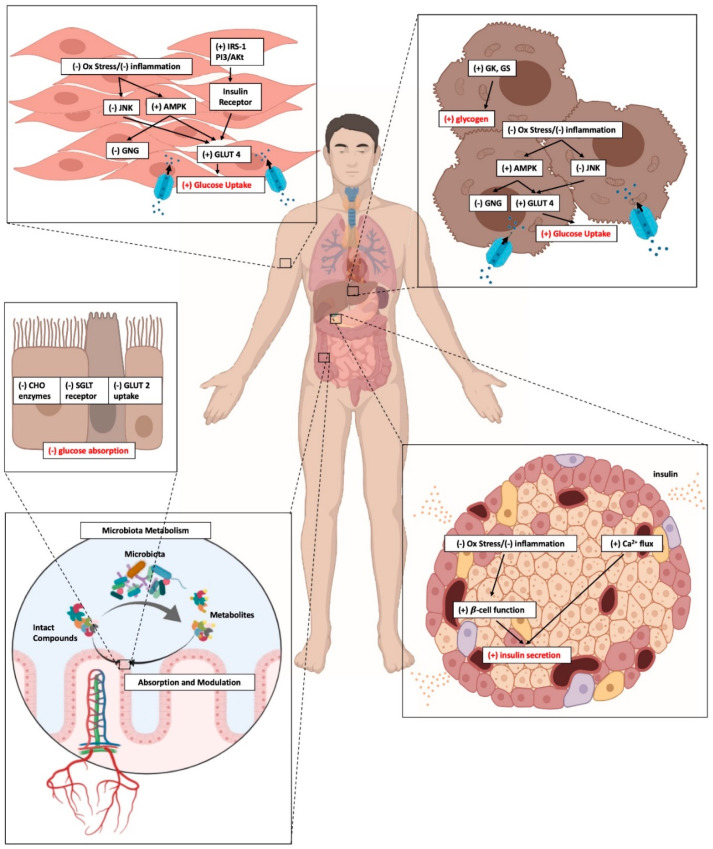
Cellular signaling pathways by which anthocyanin can modulate glucose homeostasis.

These findings are further supported by a number of preclinical studies in animals and in vitro models indicating that anthocyanins can work through several cellular signaling pathways, to achieve glucose homeostasis (Figure 2).

### 2.2. Animal, In Vitro and In Silico Studies

#### 2.2.1. Anthocyanins Acting as Promising Inhibitors of α-Amylase and α-Glucosidase Enzymes, and Glucose Transporters (GLUTs)

Carbohydrate-metabolizing enzymes are attractive therapeutic targets of T2DM, due to their key role in the balance of postprandial blood glucose. The human digestive system has different enzymes that work in synergy to completely digest dietary carbohydrates. The breakdown of polysaccharides begins with the salivary and pancreatic α-amylases cleaving the α-1,4 glycosidic bonds of starch into maltose, maltotriose and oligoglucans [48]. Afterwards, these shorter molecules are then broken down further to absorbable monosaccharides by α-glucosidases (maltase and sucrase) located in the brush-border of the small intestine [49]. Then, the glucose transporters or carriers across the small intestine will be activated.

Since the inhibition of the carbohydrate-digestive enzymes reduces the postprandial glycaemia levels, it is one of the best therapeutic strategies to counteract metabolic alterations related to hyperglycemia and T2DM, as well as to help to avoid the onset of late diabetic complications. Currently, synthetic inhibitors, such as acarbose and miglitol, are marketed and widely used as therapeutic drugs for T2DM treatment. However, they still have several side effects (e.g., hepatic injury and gastrointestinal complications) and are restricted for patients with renal insufficiency, which limits their success [50]. As a consequence, many efforts have been made in the past two decades to search for novel natural and less-toxic molecules. Many studies have demonstrated that anthocyanins or anthocyanin-rich food intake is beneficial and suppress postprandial glycaemia through the effective inhibition of α-amylase and α-glucosidase enzymes, as well as by interfering with glucose transport [51]. Specifically, previous studies have proven the ability of phenolic-rich ingredients (berry extracts, soft-fruits, grape seeds and bitter melon to inhibit the activities of pancreatic α-amylase and intestinal α-glucosidase in the gut lumen [51,52]. Other studies have shown that anthocyanin-rich fractions from blueberry [53], blackberry [54], black legumes [55], black mulberry [56], bilberry and cranberry [57], red aril fruits [58], camelina and ophia seeds [59], royle fruits [60], fruits of *Chilean berberis* species [61], peach fruit and *A. melanocarpa* fruit juice extracts [62,63], colored extracts of Saco sweet cherry [64], and anthocyanin extracts from black bean hull, black currants and black rice [65,66] exhibited higher effectiveness to inhibit α-glucosidase. Therefore, diet-induced treatments from anthocyanin-based extracts, often combined with other polyphenols, may offer a natural alternative to achieve better glycemic control in T2DM. However, despite the validity of all these studies, anthocyanins are very unstable and susceptive for degradation under physiological conditions (from the mM concentrations within the intestine, only nM concentrations reach the bloodstream). Therefore, several technologies (e.g., nanoparticles and co-microencapsulation) were developed to overcome this issue [67,68,69], and subsequently may yield the use of anthocyanins as a viable alternative for prevention/treatment in T2DM models. For example, Enache et al. co-microencapsulated anthocyanins from black currant extract and lactic acid bacteria using a gastrointestinal-resistant biocomposite [70]. They showed the α-amylase and α-glucosidase inhibitory activity of this co-microencapsulated powder of ca. 87% and 37%, respectively, indicating a high degree of protection and functionality of the anthocyanins.

In this section, the inhibitory effects and potential inhibitor selectivity of anthocyanins into the carbohydrate-digestive enzymes will be collected. Table 1 shows the available kinetic data for anthocyanins against the human carbohydrate metabolizing enzymes.

The most potent anthocyanins (IC_50_ < 20 µM (or μg/mL) targeting α-glucosidase are pelargonidin-3-rutinoside (Pg3R), cyanidin (Cyd), pelargonidin-3-glucoside (Pg3G), malvidin-3-arabinoside (M3A) and peonidin-3-arabinoside (Peo3A); whilst for α-amylase the most potent anthocyanins are Cyd, delphinidin (Dpd), malvidin (Mvd) and cyanidin-3-glucoside (C3G). This suggests a more susceptibility of the α-glucosidase and α-amylase activities to glucoside anthocyanins and non-glucoside compounds, respectively.

Previous studies have suggested that the reaction mechanism of both enzymes uses a nucleophilic and general acid/base catalysis and occurs via a double displacement mechanism characterized by oxocarbenium-ion-like transition state geometries [83,84]. In addition to the catalytic carboxylic residues positioned on opposite sides to embrace the substrate, some polar and aromatic surrounding residues (e.g., Y, R and W amino acids) provide an extensive network of H-bonds that causes conformational distortion of the substrate upon binding. Such distortion favors nucleophilic attack on anomeric carbon and subsequent cleavage of the glycosidic bond, and lowers the energetic barriers [85,86].

Recent enzymatic kinetics and in silico studies [53] determined the α-glucosidase inhibition activities of the main polyphenolic molecules present in blueberry, blackcurrant, and blue honeysuckle fruits. Despite the higher quantity of glycosidic anthocyanins, they observed a better inhibitory activity for anthocyanidins, namely Dpd, Cyd and peonidin (Peo). Molecular docking studies revealed that all three molecules bound into the active site of α-glucosidase, and the ranking of their binding affinities agrees with the order of their IC_50_ values: Dpd > Cyd > Peo. The binding mode is similar, occurring mainly via H-bonds and electrostatic interactions with polar and charged residues, and π-π stacking contacts with the aromatic residues of the binding site. Similar interactions were pointed out as relevant for the maintenance of substrate throughout the entire catalytic pathway of the human maltase-glucoamylase, recently studied with atomistic detail [87]. You and co-authors found that anthocyanin aglycones have a greater inhibitory activity (Cyd > cyanidin-3,5-diglucoside (C3,5diG)) [88]. Since during intestinal digestion the glycosidic anthocyanins are hydrolyzed by cytosolic β-glucosidase into their aglycon form, it is expectable that the glucoside compounds possess α-glucosidase inhibitory activity in vivo. Another recent study also reported a strong inhibitory activity of Cyd from *Cinnamomum camphora* fruit, and even better than that of the marketed competitive inhibitor acarbose [71]. However, other studies showed great effectiveness of glucoside anthocyanins. For example, D’Urso et al. recently reported that the anthocyanins present in black currants are very effective at inhibiting intestinal α-glucosidase (ranking of C3G > Cyd > Dpd > delphinidin-3-glucoside (D3G) > Mvd > cyanidin-3-arabinoside (C3A)) [89]. Another in vivo study also showed great effectiveness of C3R to inhibit α-glucosidase and to decrease plasma glucose levels [81].

A combined in vitro, in silico and in vivo study [78] identified the Pg3R isolated from strawberries as the most potent inhibitor against α-glucosidase (IC_50_ of 1.69 µM, Table 1). It was found that compounds with potent inhibitory activity modify the enzymatic secondary structure (namely from α-helix to β-sheet). Molecular docking analysis indicated that Pg3R bound into the binding site of α-glucosidase, establishing H-bonds with the carboxylic catalytic residues. Contrarily, the aglycone anthocyanins with poor activity, such as D3S and C3S, do not enter deeply in the active site.

In vitro and in silico inhibition experiments showed that glycosylated anthocyanins inhibit competitively porcine pancreatic α-amylase [80]. The order of the inhibition activity was C3G > cyanidin-3-rutinoside (C3R) > C3,5diG > peonidin-3-glucoside (Peo3G). The molecular docking data also indicated that all compounds bound into the active site of α-amylase, establishing H-bonds with surrounding residues. The interaction with E233 was identified as a common feature for the inhibition activity of anthocyanins. The importance of E233 was supported by a computational study of the catalytic mechanism of the human pancreatic α-amylase, in which the D197, E233 and D300 residues and two water molecules were identified as essential for catalysis [90].

Previous studies reported that Cyd and its glucosides inhibit carbohydrate-digestive enzymes by establishing covalent and/or non-covalent interactions between their hydroxyl groups and polar residues of the enzymatic active sites (by their amide, amine and carboxyl groups) [72,81,91]. The order of inhibition activity against intestinal sucrose was C3R > cyanidin-3-galactoside (C3Ga) > C3G > Cyd and C3,5diG. The great potency of C3R may be attributed to the presence of a disaccharide instead of a monosaccharide at C3.

A study of the influence of the red fruit components on α-amylase and α-glucosidase activities have identified some anthocyanins (D3G > petunidin-3-glucoside (Pet3G) > Peo3G) as potential inhibitors (even better than acarbose in the α-glucosidase assays) [92]. The obtained inhibition order, as well as many other studies, proposed that a high amount of hydroxyl groups on ring B is advantageous for the inhibition, whilst the methylation and methoxylation reduces the inhibitory activity [79,80]. However, Kaeswurm et al. observed an insignificant impact on the porcine α-amylase activity with different substitution pattern on the anthocyanin B-ring [93]. These different findings may occur due to the specific inhibitor selectivity of each enzyme. The same authors also found that the acylation of anthocyanins of black carrot with cinnamic acids improves the inhibitory effect against the porcine α-amylase [93]. This could happen due to additional dispersive contacts between the enzyme and the extra aromatic group. Matsui et al. corroborated the potent inhibitory activity of acylated anthocyanins, being sometimes better than the non-acylated counterparts [76].

The details, on an atomic level, of the mechanism by which anthocyanins inhibit carbohydrate-metabolizing enzymes are still not fully understood. However, past experimental and theoretical studies, as well as the properties and molecular structure of anthocyanins, suggest that they could act as glycomimetics inhibitors that mimic saccharide substrate structures [94]. They may inhibit both enzymes by establishing similar interactions with the key active site residues (namely, the anthocyanin-glucoside forms) or by mimicking positive-charge buildup at the transition state geometries, or both. Furthermore, the large size of some substituted anthocyanins also suggests that they may act as a cap that blocks access to the active site and/or disturb protein folding, as pointed out by fluorescence studies [78,95], which may subsequently prevent correct substrate binding for catalysis.

Regarding the enzyme activities, synergistic inhibitory effects have been observed between different anthocyanins and between anthocyanins and other phenolic compounds, when using extracts from pigmented cereals and grains [96]. Furthermore, Cyd and cyanidin glucosides (C3Ga, C3G, C3,5diG and C3R) also exhibited a synergistic effect with a low concentration of acarbose in the inhibition of sucrase, maltase and α-amylase [72,81]. This synergistic effect is particularly relevant for the development of the combined therapy for T2DM because the intake of anthocyanin-rich foods together with acarbose may lead to a lower prescribed drug dosage, and in turn lessen the adverse side effects of acarbose [72].

Several kinetic studies showed that Dpd, Cyd, Peo, C3G, malvidin-3-glucoside (M3G), Peo3G, Pg3R and C3Ga act by a mixed type of inhibition, in which they can affect both the affinity of the enzyme to the substrate and the catalytic activity of the enzyme [58,78,82]. Cyd from muscadine grapes and the anthocyanin extract of a purple-fleshed potato cultivar showed a noncompetitive inhibition against α-glucosidase and both α-glucosidase and α-amylase, respectively [88,97]. The aglycones Dpd, Cyd, Mvd and Peo from cherries showed competitive inhibition against α-amylase [79].

The inhibition of GLUTs also represents an interesting therapeutic strategy. In fact, several studies demonstrated that the reduction on the expression of these functional proteins reduced the diabetic conditions in rats [98,99,100]. Recent studies focused on the absorption of anthocyanins with glucose have associated these flavonoids to glucose transporters. In caco-2 cells, anthocyanin transportation across the cell barrier diminished upon the co-treatment with (3)*H*-2-deoxy-d-glucose in about 60%. Also, the pre-treatment of the cells with the anthocyanins led to a higher expression of GLUT-2 in caco-2 cells, suggesting that anthocyanins may modulate the metabolism of glucose not only by direct competition, but also at the gene level [101]. Another study with red wine anthocyanins, using MKN-28 cells as a model (with a high expression of GLUT-1 and GLUT-3) showed a direct competition between the anthocyanins and glucose for the transporters, translated by a reduction of anthocyanins transport upon the co-treatment with glucose. Molecular docking data also demonstrated an interesting affinity of monoglucoside anthocyanins for both GLUTs [102]. More recently, a similar approach with purple-sweet potato anthocyanins was utilized. Moreover, the complex acylated anthocyanins demonstrated to have an affinity to glucose transporters translated by a reduction of their transport in the presence of glucose on both caco-2 and MKN-28 cells [103]. Furthermore, the genetic silencing of GLUT-1 and GLUT-3 resulted in a significant reduction of anthocyanins transport [104]. In the same study, both monoglucosides and complex acylated anthocyanins interacted with the glucose transporters as demonstrated by in silico studies. Altogether, these studies demonstrated that anthocyanins can interact with glucose transporters, with low dependence on their structure. From a nutritional point of view, this may represent a great advantage to control the postprandial glycaemia, resulting in a potential strategy to improve the diabetic condition. Interestingly, not only the native anthocyanins may interact with GLUTs, but also their metabolites (discussed later), as recently demonstrated for malvidin-3-*O*-glucuronide [105].

In vivo studies also corroborated the in vitro findings [52]. The intake of anthocyanin-rich blackcurrant drinks resulted in a decrease of postprandial blood glucose, insulin and incretin after men and women were given the drinks succeeding a high carbohydrate meal [106]. The ingestion of bilberry extract by male volunteers with T2DM resulted in a significant reduction of both glucose and insulin compared with placebo [107]. Lingonberry and blackcurrant purées reduced glucose and insulin plasma levels in the first 30 min post-consumption by healthy women [108].

#### 2.2.2. Anthocyanin-Rich Functional Foods in the Prevention and Management of T2DM

Although different types of pharmacotherapeutic interventions are available for the management of T2DM, including oral hypoglycemic agents or insulin sensitizers, the ineffectiveness of the current medical treatments in the management of long-term diabetes complications confirms that the development of complementary approaches is, therefore, of utmost importance [109,110,111]. In the last years, Medical Nutrition Therapy (MNT) has become an important approach for T2DM management. In MNT, carbohydrate counting, as well as glycemic index and glycemic load, the estimation of energy and nutrients requirements or recommendation for dietary fats and cholesterol and protein intakes, are some of the main components of a healthy diet planning in T2DM patients [112,113]. In recent years, the consumption of plant-based functional foods, rich in polyphenols, has also been suggested as a potentially complementary approach in the control of several aspects of T2DM and pre-T2DM patients, mainly due to their biological properties [114,115,116,117]. Functional food is a designation related to foods that beyond the basic nutritional functions, possess potential health benefits related to the prevention of several chronic diseases, due to biologically active ingredients [118]. Different types of foods can be included in the functional foods category, ranging from foods with functional properties, like fruits, vegetables or whole grains, to modified foods in which for instance sugars are replaced by non-nutritive sweeteners and to fortified foods, like those enriched with phytochemicals or nutrients [119]. Regarding polyphenol-rich functional food intake, there is a substantial body of in vitro, in vivo, and epidemiological evidence suggests a positive health outcomes in diabetic patients [120,121,122,123]. Within polyphenols, anthocyanins appear to be one important class supporting the prevention and treatment of T2DM [124].

Over the past years, numerous studies have demonstrated that anthocyanins can exert a beneficial effect on T2DM by regulating different signaling pathways in several organs and tissues, such as liver, pancreas, kidney, adipose, skeletal muscle and brain [51]. Anthocyanins may lower blood glucose by improving insulin resistance, protecting β-cells, increasing the secretion of insulin, reducing digestion of sugars through α-amylase and α-glucosidase inhibition, reduce the oxidative processes, decrease the inflammatory response, upregulating glycolysis gene expression, downregulating gluconeogenesis gene expression and activating AMP-activated protein kinase (AMPK) [76,125,126,127]. As previously mentioned, carbohydrate enzymatic inhibition by common glycosides, di-glycosides or acylated anthocyanins, undoubtedly contributes to anthocyanins antidiabetic effects [128,129]. The highest inhibitory activity was reported to acylated anthocyanins [76], which might be due to its greater stability in the gut [130]. Anthocyanins fortification of cereal-based food products has also been shown to be an alternative way to produce functional food products for T2DM management and control. Zhou et al., showed that starch digestion rates of bread dough fortified with anthocyanins extract from black rice were reduced by 12.8% when 1% of anthocyanin extract was added. Carbohydrate digestion rate dropped further to 20.5% when the number of anthocyanins reached 4%. This suggests a lower insulin demand, potentially improving the blood glucose control and providing extra health benefits, due to anthocyanins antioxidant potential [131]. In vitro studies to evaluate the potential of anthocyanins in insulin resistance have been conducted in hepatocytes (HepG2 cells), human 3T3-L1 adipocytes, rat liver cells (H4IIE cells), muscle cells (L6 myotubes), rodent pancreatic β-cells and satellite cell [126,128,129,132,133,134,135]. Additionally, a vast number of in vivo protective activities of anthocyanins rich extracts against insulin resistance and obesity conditions have been reported. These extracts were found to effectively ameliorate insulin resistance condition, increase insulin sensitivity, decrease body weight gain and lipids accumulation [136,137,138,139].

**Figure 3:**
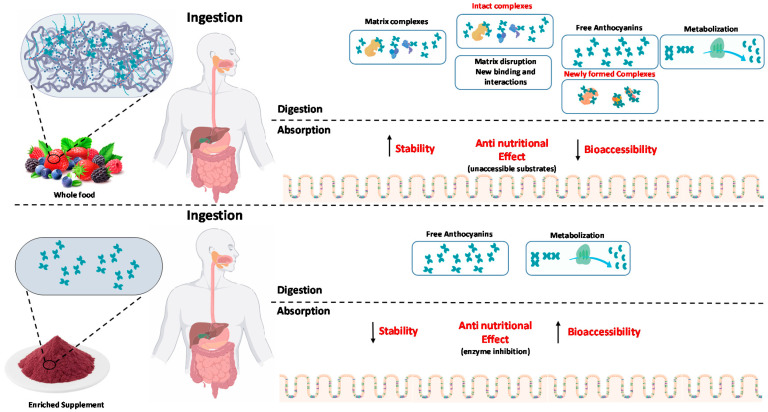
Impact of anthocyanin consumption within food matrix and as a supplement (individual compounds).

Despite several in vitro and in vivo lines of evidence, it is not clear the impact of delivering anthocyanins in whole plant foods, compared with individual functional ingredients, as supplements (Figure 3). The main difference is the presence of other natural biomolecules, such as proteins and cell-wall polysaccharides, that co-exist with anthocyanins in most plant-based food products (vegetables, fruits, fruit juices). When cells are ruptured, during mastication and/or processing (boiling, pureeing, homogenization, juicing or canning), anthocyanins and biopolymers may interact, forming complexes through non-covalent forces or covalent bonds [140,141,142]. The non-covalent interactions, mainly mediated by hydrogen bonds, van der Waals forces, hydrophobic interactions and electrostatic interactions, are known to exert heavy impacts on the extractability, stability, functional, nutritional, biological and sensorial properties of these combined systems. Anthocyanin-polysaccharide non-covalent complexes have been shown to improve anthocyanins thermostability [143], increase their stability under in vitro gastric and small intestinal digestion [144,145] or enhance anthocyanins color stability [146]. Nutritionally, these interactions may also play an important role in the digestive tract of humans, regarding the metabolism of both anthocyanins and polysaccharides [142,147]. Recently, the antidiabetic activity of polysaccharides has also been reported [148,149,150]. These effects were mostly attributed to enzyme inhibition related to starch digestion and mechanisms related to inflammatory factors and oxidative stress, exerting favorable effects on glucose homeostasis and reducing insulin resistance. Due to this, there has been considerable interest in understanding and exploiting the functions and health benefits of the whole food systems as these conjugates may have the improved health attributes that cannot be achieved using individual components. However, there is very few information related to the polyphenols-polysaccharide bound complexes (both non-covalently and covalently bound) and their anti-diabetic activity, with these works being essentially focused with other polyphenols classes, rather than anthocyanins. Guo et al., reported an IC_50_ for α-amylase inhibition of 6.11 and 5.75 mg/mL and an IC_50_ for α-glucosidase inhibition of 9.01 and 4.66 mg/mL, for corn silk polysaccharides and flavonoids-corn silk polysaccharides, respectively [151]. Regarding plant proteins, Jiang et al., showed that naturally occurring protein-bound anthocyanin complexes from purple sweet potato were able to ameliorate hyperglycemia, through the regulation of the hepatic glucose metabolism in high-fat diet/streptozotocin-induced diabetic mice [111]. The results showed that the administration of anthocyanin-protein complexes improved the glucose tolerance and lipid metabolism, decreasing the oxidative stress and liver damage by regulating the expression of proteins in the glucose transport system and then increasing glycolysis and decreasing gluconeogenesis in the liver.

### 2.3. The Role of Anthocyanins Targeting Glycogenolysis

Another pharmacological strategy against T2DM focuses on retarding glycogenolysis. Previous studies proposed that anthocyanin-rich foods also regulate this metabolic cascade by interfering with the activity of key enzymes, such as Glycogen phosphorylase (GP) [75,152,153,154]. GP catalyzes the rate-limiting step of glycogen breakdown, releasing glucose-1-phosphate. After this, phosphoglucomutase shifts the position of the phosphate in the sugar, making it suitable to fulfill the energetic requirements of the organism [155]. GP is an allosteric enzyme that changes between active (GPa) and tense (GPb) conformation states. Recently, the GP catalytic mechanism has been assessed at the atomistic level, and the characterized transition state geometries could be now used in structure-based drug design studies [156].

Jakobs et al. studied the inhibition activity of several classes of flavonoids targeting GP [75]. As to anthocyanins, they obtained an inhibitory order of Cyd > Dpd > Peo > Pgd > Mvd (as seen in Table 1). They also suggested that the substitution pattern of the B ring of anthocyanidins influences their inhibitory effect. For example, the most potent inhibitors, Cyd and Dpd, have vicinal hydroxyl groups in the B ring and their inhibition activity occurs in the same low range (IC_50_ = 3 µM, Table 1). However, compounds without one of the vicinal groups, such as Pgd, had a significantly reduced inhibitory activity (IC_50_ = 43.6 µM, Table 1) whilst the replacement of some hydroxyl groups by methoxy groups (exemplified by Mvd) decreases the inhibitory activity even more (IC_50_ > 50 µM, Table 1).

Curiously, the most abundant anthocyanidin in nature, Cyd, is a potent inhibitor for the three human carbohydrate metabolizing enzymes: α-glucosidase, α-amylase and GP. In general, anthocyanins are able to ameliorate disorders in glucose metabolism and can be more explored as key natural-based inhibitors in the prevention and treatment of T2DM.

### 2.4. The Interplay between Anthocyanins and Gut Microbiota

The biological effects of the compounds will mainly depend on their interactions with the colonic environment. Such a path depends heavily on the microbiota, and its action on the biotransformation of anthocyanins will play a key role in determining their exerted biological effects [157]. Besides this, anthocyanins may also modulate microbiota, exerting indirect effects on different pathological conditions [158].

#### 2.4.1. Anthocyanins and Gut-Derived Metabolites and Their Antidiabetic Potential

When assessing the metabolization of natural compounds, it is important to have present that of several factors may influence the action of the enzymatic and bacterial machinery, namely, inter-individual differences of the subjects used in the studies or the food matrix represent important examples of factors that may modulate the enzymatic and bacterial action upon these compounds, in particular anthocyanins. In the study of Czank et al., the human metabolism of cyanidin-3-*O*-glucoside (C3G) was assessed using a ^13^C-tracer [32]. The authors found that upon the consumption of 500 mg of ^13^C-labeled C3G, the recovery of ^13^C in urine, blood and faeces on the eight subjects analyzed, varied from 15.1 to 99.3%. In a recent study, it was found that the food matrix acted as a shield against the enzymatic degradation of anthocyanins upon simulated gastrointestinal digestions [103]. Such facts contribute to a complex network of metabolic fates, leading to a wide range of natural compounds metabolites, particularly in the case of anthocyanins.

Although the complete screening and understanding of the bacterial and enzymatic catabolism of the different dietary anthocyanins are far from being achieved, some food sources are by now well characterized. A recent study showed that one of the anthocyanins in the extract of *Aronia melanocarpa*, C3G, appeared in the plasma of human subjects 30 min after consumption; however, plasma protocatechuic acid peaked as early as 1 h [159]. Other anthocyanin metabolites were detected in plasma after a longer period, such as hippuric acid 3-(4-hydroxyphenyl)-propionic acid and Peo3Ga. In the same study, the authors analyzed urine samples and detected the same metabolites as in plasma plus intact anthocyanins (such as C3G, C3Ga, C3A) and other metabolites (such as ferulic acid and 3,4-dihydroxyphenylacetic acid). Despite the interindividual differences, the authors suggested that the majority of the metabolites were derived from the main anthocyanin of Aronia extract, C3Ga, since it was not detected in any of the plasma samples.

Anthocyanins from blackberry are also extensively metabolized upon ingestion. Blackberry is characterized by the presence of the main anthocyanin, C3G [160]. The consumption of 200 mg of blackberry resulted in the presence of intact C3G and metabolized forms in urine after 2 h in the urine of human volunteers. The total urinary excretion of blackberry anthocyanin metabolites was about 0.2%. The metabolites were identified as being mainly anthocyanin monoglucoronides, and in a lower extent, methylated and sulfoconjugated derivatives [28]. Similarly, anthocyanins main metabolites of boysenberry identified in human urine after consumption were monoglucoronides of peonidin, cyanidin and pelargonidin [161]. In another study, with blueberry, monoglucoronides and methylated anthocyanins were found in urine [162].

Very recently, 63 blueberry anthocyanin metabolites were identified and quantified in human plasma. This included the presence of cinnamic acids, benzoic acids, phenols, hippuric acids, phenylacetic acids and flavonols after 2 h post-consumption [163]. The same authors had previously identified similar metabolites after blueberry consumption, including phase II metabolites, namely, catechol-*O*-sulfate, dihydrocaffeic acid 3-*O*-sulfate, isoferulic acid 3-*O*-β-d-glucuronide [164].

In a recent clinical trial, red wine and Port wine were given to volunteers, and after the consumption of the wines, anthocyanins were detected in their intact forms in both plasma and urine samples, but the glucuronylated metabolites of peonidin and malvidin were the two main derivatives detected after both red wine consumptions [25]. Furthermore, evidence that red wine anthocyanins are also extensively metabolized came up more than a decade ago [165,166].

Elderberry anthocyanin catabolism by three gut microbiota bacteria species was recently explored. Enterobacter cancerogenous, *Bifidobacterium dentium* and *Dorea longicatena* degraded the anthocyanins, but in interestingly different metabolic pathways [167]. About 30 metabolites, including cinnamic and benzoic acids, and also intact anthocyanins were detected in the three cell culture samples. Indeed, a previous study demonstrated different metabolic patterns for the degradation of C3G when incubated with different bacterial strains and with human faecal slurries [168].

Red cabbage was given to healthy volunteers and metabolites were explored in urine and plasma after intake of red cabbage products. Apart from the eighteen native anthocyanins, the presence of twelve phase II metabolites of cyanidin, including methylated, glucuronidated and sulfated forms, were found [169]. Interestingly, red cabbage contains complex acylated anthocyanins, and this may influence the range of metabolization of these compounds. Furthermore, other few studies with eggplant, purple sweet potato and purple carrot acylated anthocyanins regarding the consumption of acylated anthocyanins were performed, but in all cases only the native forms were explored and detected, remaining unclear the clear fate of this kind of anthocyanins [170,171,172].

**Figure 4:**
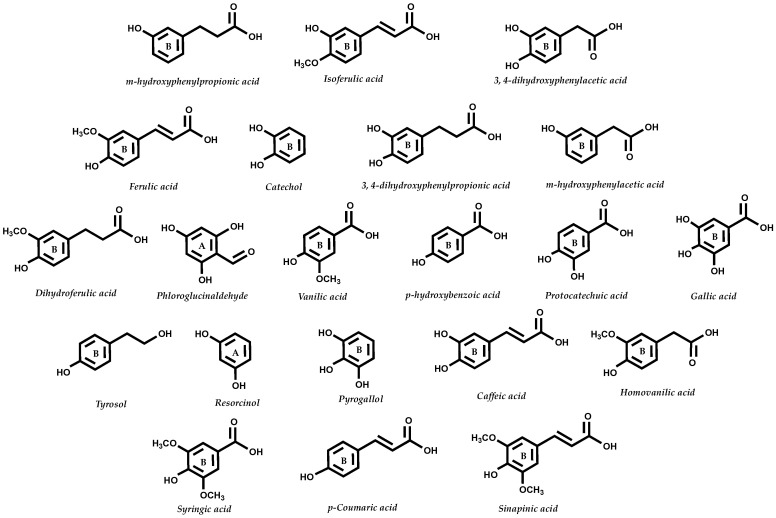
Main gut anthocyanin metabolites. Metabolites can be formed by enzymatic/microbiota action, and result from the breakdown of the C6-C3-C6 structure. Thus, metabolites can be either derived from ring A or B of the parent anthocyanin structure.

The vast majority of the food sources explored have high contents of monoglucosides anthocyanins such as C3G, the most abundant anthocyanin in nature, which may explain the consistency in the formation of the known metabolites. Figure 4 summarizes the main gut metabolites derived from anthocyanins.

These anthocyanin-rich foods have been linked to antidiabetic properties in vitro and in vivo. T2DM a chronic exposure to high glucose levels cause the irreversible failure of pancreatic β-cells via increased oxidative stress. Recently, it was shown that in pancreatic β-cells the anthocyanin-rich fraction of *Aronia melanocarpa* presented a scavenging effect on the intracellular oxygen species, revealing to be an interesting therapeutic strategy [173]. In another recent study, the consumption of chokeberry (*Aronia melanocarpa*) and dried jujube by C57BL/6 J mice during a 10-week period, changed the hepatic protein expression of the insulin receptor, insulin receptor substrate 1, phosphoinositide 3-kinase, Akt, and catalase, which are associated with insulin resistance. This led to a consequent improvement on insulin resistance, according to several indicators, such as serum insulin level, fasting blood glucose level, or oral glucose tolerance test value [174]. In another study, diabetic Wistar rats were fed with *Aronia melanocarpa* extract for 16 weeks. The results showed an immunomodulation of key cytokines in insulin-deficiency diabetes, such as TNF-α and IFN-γ, reducing the inflammatory status of the diabetic rats [175].

Blueberry anthocyanins were linked to the protection of retinal cells from diabetes-induced oxidative stress and inflammation via the regulation of Nrf2/HO-1 signaling [176]. These anthocyanins also showed to prevent the increased blood glucose on diabetic rat models [177]. Another study showed that red raspberry intake by diabetic mice modulates the oxidative stress and increases the antioxidant defenses [178].

Either in vivo or in vitro and regardless of the food source, these compounds demonstrated the capability to act through a multi-target pathway, modulating aspects, such as transcription factors, pro- and anti-inflammatory cytokines, insulin and glucose metabolism, and lipid metabolism, resulting in antidiabetic agents [38,46,179,180,181,182].

Regardless of the well-explored antidiabetic effects of the different anthocyanin food sources, it is important to assume that the observed effects may not be totally attributed to the anthocyanins but rather to their metabolites. Indeed, such metabolites derived from anthocyanins have also been shown to have biological properties against diabetes. The effect of protocatechuic acid (PCA) in the chronic inflammation in visceral adipose tissue (VAT) that may lead to insulin resistance and T2DM was evaluated [183]. The results showed that this metabolite restored the lack of insulin response in the obese-VAT individuals, by increasing the phosphorylation of key factors for insulin sensitivity, such as IRS-1 and Akt, which may be due to the inhibition of protein tyrosine phosphatase 1B activity, a well-known insulin signaling regulator. PCA also diminished the overall inflammatory state of the obese individual by lowering the nuclear factor kappa B (NF-κB) pathway. Hence these results suggest an important role of this gut-derived metabolite against inflammation and insulin resistance, two enhancers of diabetes. In another study, protocatechuic acid showed to reduce the lipid profile and HDL-C levels to near control after treatment of diabetic rats with PCA [184]. The metabolite was also found to stimulate the insulin signaling pathway by increasing glucose transporters (GLUT4) translocation with a consequent better glucose uptake in human visceral adipocytes [185]. The metabolic origin of hippuric acid is derived from the action of microbiota, causing the degradation of phenolic compounds to benzoic acid followed by conjugation with glycine moiety in the liver [186]. As stated before, the rise in the concentration of this metabolite in urine and blood is associated with the consumption of anthocyanins [32,164,187,188]. Recently, it was observed that fasting serum hippuric acid levels were higher after the consumption of bilberry. Interestingly, in the same study, this metabolite was not detected upon the consumption of other anthocyanin-rich foods, such as strawberries, raspberries, and cloudberries, as well as in the control group. Moreover, the authors observed that the increase in hippuric acid levels was associated with an improvement of fasting plasma glucose concentration and insulin secretion in individuals with the metabolic syndrome with the risk of developing T2DM [189].

Ferulic acid is also a common metabolite found upon the consumption of anthocyanins. This metabolite is associated with restored blood glucose, serum insulin, glucose tolerance, and insulin tolerance to normal range in diabetic rats [190]. Very recently, diabetic rats treated with ferulic acid for 8 weeks demonstrated a reduction of hyperglycemia-injured kidney through the regulation of oxidative aggressions, inflammation and autophagy [191]. Other studies, performed in similar animal models of diabetes, are consistent with the efficacy of this metabolite either through a direct effect on glucose metabolism or through the modulation of genetic factors responsible for the development of diabetes. A study demonstrated that ferulic acid significantly increased the expression of two important enzymes for the protection of oxidative stress in obese rats (heme oxygenase-1 and glutathione-S-transferase), while reducing the expression of other enzymes normally involved in tissue injury or necrosis [192].

Also, in diabetic rats, caffeic acid attenuated the diabetic kidney disease via modulation of autophagy, after a 12-week treatment. The authors suggested a mechanism of action involving the suppression of key authophagic miRNAs [193]. In a HepG2 insulin-resistance cell model, caffeic acid demonstrated antidiabetic effects through the attenuation of insulin-resistance and modulation of glucose uptake. The expression of IRS-1, Akt, PI3K, and GLUT-4 showed significant up-regulation in the case of phenolic acids treated samples [194].

Other phenolic acids that appear in plasma and urine after the consumption of anthocyanin-rich foods have also been extensively studied for their role in diabetes, such as ellagic, syringic, synaptic, or coumaric acids [195]. Similarly to the previously discussed, several mechanisms have been described for the antidiabetic actions of these compounds, including the regulation of glucose transporters translocation, modulating glucose metabolism, improving insulin resistance and modulating gene factors involved in the susceptibility for diabetes development [196,197,198,199].

Not only the direct products of anthocyanin breakdown present anti-diabetic effects. Enzymatic phase I and II metabolites of anthocyanins have also been hypothesized as having potential against diabetes and related disorders [16,105,200].

#### 2.4.2. Gut Microbiota Modulation by Anthocyanins

Host and gut microbiota (GM) live in a synergistic way, as a result of thousands of years of co-existence and evolution. Therefore, the microbial environment of the gastrointestinal tract has important roles in the homeostasis of the whole host organism. The role of GM in health and several diseases have been extensively explored through the years, and a relationship between the imbalance of GM and type 1 and 2 diabetes is nowadays, a fact [201,202,203].

The modulation of gut microbiota is highly dependent on dietary habits. Nutrients can act both directly or indirectly with the microorganisms in order to promote its growth or inhibition and normally, healthy diets rich in polyphenols are associated with better microbiota composition [204]. In this matter, anthocyanins may also act as modulators of gut microbiota, and although limited research has been performed, evidence supports the capability of anthocyanins to change the microbial environment of the gastrointestinal tract. Zhang et al. verified that purple sweet potato anthocyanins were able to modify the human intestinal microbiota in vitro. The study was conducted by monitoring bacterial populations and short-chain fatty acids at different time points, and the results showed that the anthocyanins induced the growth of *Bifidocterium* and *Lactobacillus* spp. and inhibited the growth of *Bacteroides-Prevotella* and *Clostridium histolyticum* [205]. In a more recent study, anthocyanins from the same source had demonstrated specific effects on the modulation of different gut bacteria strains. After simultaneous incubation, the compounds were able to induce the proliferation of *Bifidobacterium bifidum*, *Bifidobacterium adolescentis*, *Bifidobacterium infantis* and *Lactobacillus acidophilus*, and they inhibited the growth of *Staphylococcus aureus* and *Salmonella typhimurium*, suggesting that purple sweet potato anthocyanins might have prebiotic-like activity [206]. The consumption of different berries rich in anthocyanins led to a change in gut bacterial populations in C57BL/6 mice model. Animals consuming Concord grape showed the largest expansion of Actinobacteria from 2% to 18%. Blueberry and blackcurrant supplementation was associated with significant shifts in the fecal bacterial profiles that simultaneously increased populations of obligate anaerobes Bacteroidetes (from 7% to 10–12%) and Actinobacteria (from 2% to 9–15%) [207]. Strawberry supplementation increased the abundance of beneficial bacteria Bifidobacterium in mice [208]. In other recent work, the main anthocyanin from *Lycium rythenicum Murray* (petunidin-3-*O*-[rhamnopyranosyl-(trans-p-coumaroyl)]-5-*O*-[β-d-glucopyranoside]) was able to reverse the dextran sodium sulfate-induced colitis decrease of Porphyromonadaceae, Rikenellaceae and Prevotellacea in mice [209]. Black raspberry anthocyanins enhanced the growth of *Eubacterium rectale*, *Faecalibacterium prausnitzii* and *Lactobacillus*, and inhibited the growth of *Desulfovibrio* spp. and *Enterococcus* spp. [210]. A randomized clinical trial showed that interventions with both red wine and dealcoholized red wine increased the fecal concentration of Bifidobacterium, Enterococcus and Eggerthella lenta, compared to gin intervention and baseline [211]. Also, in a recent study, blackberry anthocyanin-rich extract was found to modulate gut-microbiota composition. Blackberry extract increased Pseudoflavonifractor when the animals were fed with the control (low fat) diet and increased Oscillobacter independently of the diet fat content [212].

The interplay between anthocyanins and gut microbiota leads to a network of changes that can be used to prevent or modulate several pathologies, as already observed. Either by the metabolizing capability of gut microbiota to biotransform anthocyanins in new structurally different bioactives or by the modulation capability of native anthocyanins to change levels of specific gastrointestinal bacteria populations, this interplay may provide an important strategy against diabetes and related diseases from a nutritional perspective.

## 3. Conclusions

**Figure 5:**
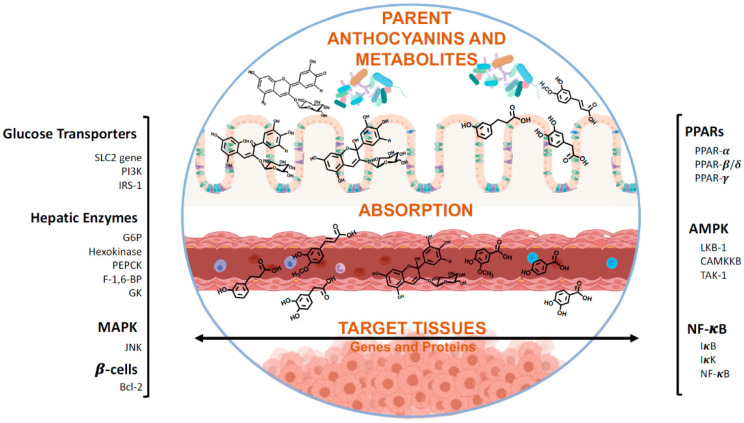
Target proteins and genes of anthocyanins and their metabolites with antidiabetic activity. After reaching the gastrointestinal tract, anthocyanins may be directly absorbed or metabolized. Once in the blood stream, they may be absorbed by target tissues of further transformed in kidney or liver. Nevertheless, upon reaching their targets, these compounds can act as antidiabetic agents at different levels, such as glucose transport, the hepatic machinery or modulation of β-cells, and on different metabolic pathways, like Mitogen-activated protein kinase (MAPK), AMP-activated protein kinase (AMPK) or nuclear factor kappa B (NF-κB), or even act on the lipid metabolism level by modulating peroxisome proliferator-activated receptors (PPARs). SLC2: Facilitative GLUT transporter family; Pi3K: Phosphoinositide 3-kinases; IRs-1: Insulin receptor substrate 1; G6P: Glucose 6-phosphatase; PEPCK: Phosphoenolpyruvate carboxykinase; F-1,6-BP: Fructose 1,6-bisphosphatase; GK: Glucokinase; JNK: c-Jun N-terminal kinase; Bcl2: B-cell lymphoma 2 proteins; LKB-1: liver kinase B1; CAMKKB: calcium/calmodulin-dependent protein kinase 2; TAK-1: Transforming growth factor beta-activated kinase 1.

Currently, synthetic carbohydrate-digestive enzyme inhibitors are marketed and widely used as therapeutic drugs for T2DM treatment. However, due to their several side effects (e.g., hepatic injury and gastrointestinal complications) and limitation to a sub-group of the population, many efforts have been made to determine the dietary recommendation that could have the same effects. Individual anthocyanins or anthocyanin-rich food may suppress the postprandial glycaemia through the effective inhibition of α-amylase and α-glucosidase enzymes, as well as by interfering with glucose transport, glycogenolysis and lipid metabolism by different molecular pathways (Figure 5).

Some studies highlight the importance of glucose on anthocyanin structure, others assign a more potent activity to the aglycone forms, and there are also examples of the inhibitory potential of acylated anthocyanins. During gastrointestinal digestion the monomeric or acylated anthocyanins these may be converted, at least in part, into their aglycon form and metabolized. For example, M3G is converted into malvidin-3-*O*-glucuronide by Uridine 5′-diphospho-glucuronosyltransferase (UGT). The same metabolite is formed with malvidin-3-*O*-coumaroylglucoside. This point may render the glycosylation and acylation pattern a hugely important in the physiological stability and modulation of enzymatic activity during digestion, rather than in the antioxidant or anti-inflammatory effect.

Both the interaction with endogenous biomolecules and interaction with exogenous biomolecules have a huge impact. Therefore, diet-induced treatments from anthocyanin-based extracts, often combined with other polyphenols, may offer a natural alternative to achieve better glycemic control in T2DM. Considering the beneficial properties of anthocyanin-rich functional foods, it seems that diet planning based on these healthy foods may constitute an effective strategy for the management of various aspects of diabetes and for the promotion of health in T2DM patients, with anthocyanins-rich foods becoming not only food products, but also therapeutic agents. However, the impact of non-covalent and covalent interactions between anthocyanins and other plant biomolecules, such as proteins or polysaccharides in whole plant anthocyanin-rich foods and how this affects the anti-diabetic activity of anthocyanins, remain unclear. Thus, more studies are needed to elucidate how exactly anthocyanin-biopolymer complexes can contribute to T2DM management, in view to optimize food processes for functional benefits. Although the mechanistic studies are fundamental to understand the impact of food matrix for the accessibility, several other factors may influence the action of the phase I and II enzymatic machinery and bacterial activity, namely, inter-individual differences of the subjects. Therefore, when the big step towards clinical trials is taken the complexity of the whole scenario rises. Besides the huge impact of microbiota on anthocyanin degradation also the reciprocal may occur, being anthocyanins able to act as modulators of gut microbiota.

The nutritional impact of a bioactive should be considered as a long-term chronic consumption and not in an acute pharmaceutical practice.

## Figures and Tables

**Table 1:** IC_50_ values (in µM) for anthocyanins against the human α-glucosidase, α-amylase and glycogen phosphorylase (GPa and GPb forms) enzymes. *—values obtained in assays with porcine pancreatic α-amylase; **—values in μg/mL.

Anthocyanin	α-Glucosidase	α-Amylase	GPa/GPb
cyanidin (Cyd)	5.293 [71]; 1420 ± 250 [72] (sucrase)	0.5 ** [73]; 380 ± 10 [72,74]	3.0 [75]/9.0 [75]
delphinidin (Dpd)		0.5 ** [73]	3.1 [75]/10.7 [75]
peonidin (Peo)	200 ± 4.1 [74]		25.1 [75]/17.6 [75]
malvidin (Mvd)		16.4 ** [73]	>50 [75]/>50 [75]
pelargonidin (Pgd)	60 [76]		43.6 [75]/6.2 [75]
cyanidin 3-glucoside (C3G)	25550 [77]; no inhibitory effect [71]; 1025.32 [78]; 970 ± 50 [72]	2.6 ** [73]; 180 ± 20 [79]; 24 ± 3 * [80]; 300 ± 10 [72,74]	
cyanidin 3-galactoside (C3Ga)	± 50 (sucrase) [81]; 250.2 (sucrase) [81]; 2300 [81] (maltase)	>1000 [72,74]	
cyanidin 3-rutinoside (C3R)	608.69 [78]; no inhibitory effect [71]	200 ± 24 [79]; 31 ± 7 * [80]	
cyanidin-3-arabinoside (C3A)		56.9 ** [73]	
cyanidin 3-sambubioside (C3S)	>4000 [78]; 3190 [77]		
delphinidin-3-glucoside (D3G)	142.68 [78]	41.8 ** [73]	
delphinidin-3-arabinoside (D3A)	22.7 [78]		
delphinidin-3-rutinoside (D3R)	69.82 [78]		
delphinidin-3-galactoside (D3Ga)	148.2 [78]		
delphinidin 3-sambubioside (D3S)	>4000 [78]		
pelargonidin-3-rutinoside (Pg3R)	1.69 [78]		
pelargonidin-3-glucoside (Pg3G)	10.35 [78]		
petunidin-3-arabinoside (Pet3A)	81.47 [78]		
petunidin-3-glucoside (Pet3G)	158.65 [78]		
petunidin-3-galactoside (Pet3Ga)	234.4 [78]		
malvidin-3-arabinoside (M3A)	11.32 [78]		
malvidin-3-glucoside (M3G)	116.77 [78]	675 ± 73 [79]; 44 ± 4 * [82] (Ki)	
malvidin-3-galactoside (M3Ga)	139.64 [78]		
peonidin-3-arabinoside (Peo3A)	18.75 [78]		
peonidin-3-glucoside (Peo3G)		75 ± 7 * [80]	
peonidin-3-galactoside (Peo3Ga)	143.31 [78]		
cyanidin-3,5-diglucoside (C3,5diG)	>2000 [72,74]	40 ± 7 * [80]; >1000 [72,74]	
